# Photobiomodulation therapy retarded axial length growth in children with myopia: evidence from a 12-month randomized controlled trial evidence

**DOI:** 10.1038/s41598-023-30500-7

**Published:** 2023-02-27

**Authors:** Lei Zhou, Liyang Tong, Ying Li, Bruce T. Williams, Kaikai Qiu

**Affiliations:** 1 Department of Optometry, Ningbo Eye Hospital, Ningbo, 315000 Zhejiang Province China; 2 Department of Ophthalmology, The First People’s Hospital of Xuzhou, The Affiliated Xuzhou Municipal Hospital of Xuzhou Medical University, Xuzhou, 221005 Jiangsu Province China; 3 Xuzhou Medical University, Xuzhou, 221005 Jiangsu Province China; 4 Seattle Vision Care Center, Seattle, WA 98101 USA; 5 Tianming Ophthalmology and Optometry Clinics, Dali, 671099 Yunnan China; 6 Airdoc MPC Co, Ltd, F4, No.2 North Road, Haidian District, Beijing, 100081 China

**Keywords:** Environmental sciences, Environmental social sciences, Biomarkers, Diseases, Health care, Medical research, Optics and photonics

## Abstract

To determine whether photobiomodulation (PBM) therapy can retard ocular axial length (AL) in children with myopia. A randomized controlled clinical trial was conducted on two consecutive cohorts of 50 eligible children aged 8–12 years with ≤ − 0.75 Diopter (D) of spherical equivalent refraction (SER). Participants were randomly assigned to the intervention group (n = 25) and treated with PBM therapy or the control group (n = 25) and treated with single vision spectacles only. At the 12-month follow-up, the changes in AL and cycloplegic SER from baseline were both compared between the two groups. In addition, the subfoveal choroidal thickness (SFChT), anterior chamber depth (ACD), and central corneal refractive power (CCP) were analysed at the 3-, 6-, 9-, and 12-month follow-ups, respectively. Among the 50 children, 78% were included at the final follow-up, with a mean age of 9.7 ± 1.5 years and a mean SER of − 2.56 ± 1.70. The mean difference in AL growth between the two groups at 12 months was 0.50 mm (PBM vs. Control, − 0.02 mm ± 0.11 vs. 0.48 mm ± 0.16, *P* < 0.001), and the mean difference in cycloplegic SER at 12 months was + 1.25 D (PBM vs. Control, + 0.28 D ± 0.26 vs. − 0.97 D ± 0.25, *P* < 0.001). There were no significant differences in any of the other parameters (including SFChT, ACD, and CCP) between the two groups at any time point. PBM therapy is an effective intervention for slightly decreasing the AL to control myopia in children.

*Trial registration*: Chinese Clinical Trial Registration Number: ChiCTR2100043619. Registered on 23/02/2021; prospectively registered. http://www.chictr.org.cn/showproj.aspx?proj=121302.

## Introduction

Sufficient outdoor time is a widely accepted method for preventing myopia^1^. High levels of ambient lighting could retard form-deprivation myopia in monkeys^2^. The change in ocular axial length (AL) involves a tremendous change in the light environment, which can be considered to play an important role in the onset and progression of myopia. The difference between indoor and outdoor light environments, such as the intensity and wavelength of electronic lighting equipment, may be a cue for myopia control as environmental interventions.

Fortunately, some clinical trials have discovered a novel and intriguing intervention—which has many different names, e.g., low-level laser therapy (LLLT)^3^, low-intensity, long-wavelength red light^4^, or repeated low-level red light (RLRL)^5^—to control myopia in children with approximately two 3-min sessions of therapy every day^3–8^. Those studies have suggested that two sessions of therapy with repeated low-intensity, single-wavelength (650 nm) red light for a total of 6 min daily could significantly retard AL) growth. Moreover, the recently reported maximum decrease in AL was 1.07 mm, which was observed in a 5-year-old high myopia patient (from baseline − 8.50 D to − 5.50 D after 10 months of red light therapy) on social media^9^.

The first well-designed randomized controlled study also reported that this special red light therapy could shorten the AL to a mean value of − 0.06 mm (74 children)^3^ after 6 months compared to the single-vision spectacles (SVS) group, who exhibited a mean AL increase of 0.23 mm (74 children), as well as the orthokeratology group, who exhibited a mean AL increase of 0.06 mm (81 children). Thus, all of the above findings on decreased ALs were quite opposite to the traditional concept that the elongated eyeball was irreversible. Although some evidence has demonstrated that PBM therapy only slows AL growth^5^ with a rebound effect^6,7^, it could be a promising pantheon for light-based therapeutic intervention.

Additional evidence regarding the effect of PBM therapy on shortening the AL in myopia came from the authorized PCT patent WO2020244675A112 with a disclosure date of 2020. In the patent, a similar phenomenon was observed, including a decrease in AL for 84 myopic children (6–23 years old) for several months (one child underwent therapy for 3 months, the other 83 children underwent therapy for 6 months) after PBM therapy twice a day.

Previously, we published a retrospective cohort study^4^ of 105 myopic children, and we found that their AL was decreased by − 0.06 ± 0.19 mm for 9 months after PBM therapy. To overcome the limitations of previous studies that lacked control groups, the high possibility of long-term observation of the tendency of AL growth in children with myopia, and AL circadian rhythm fluctuations^10^, we conducted the current prospective study to examine whether the AL of individuals with myopia might be decreased extent after 12 months of repeated PBM treatment twice a day.

## Methods

### Study design

This 12-month clinical trial was designed to be a randomized, controlled, parallel study with follow-up visits every 3 months. Potentially eligible children were recruited from the hospital after a screening visit.

The trial was designed to begin in February 2021 and end in June 2023. All subjects were enrolled at the Optometry Center of Ningbo Eye Hospital. Our study and protocol conformed to the principles of the Declaration of Helsinki and were approved by the ethical committee of Ningbo Eye Hospital (Number: 2020xjx-012).

### Eligibility criteria

The inclusion criteria were as follows: (1) the subject's guardian agreed to participate in the study and signed a written informed consent form; if the subject could express his or her willingness to participate in the trial, the subject's consent was also obtained; (2) age 3–16 years old (including the boundary values); there were no restrictions regarding gender; (3) the cycloplegic refraction with either spherical equivalent refractive (SER) was ≤ − 0.50 D ~ − 8.00 D (including the boundary values), and the total astigmatism was ≤ 2.50; (4) spectacle-corrected monocular VA was 0.0 logMAR or better; and (5) willing to wear SVS throughout the trial.

The exclusion criteria were as follows: (1) patients who intended to use atropine (including 1% high concentration or 0.01% low concentration); (2) patients wearing peripheral defocus spectacles or duo-focal soft contact lenses; (3) patients with eye diseases such as glaucoma or retinal lesions; (4) optic media lesions (e.g., central thick corneal scars, cataract); (5) patients with optic nerve dysfunction; (6) patients with amblyopia; (7) research physicians determined that the subject was not eligible for other reasons. After screening, the participants were selected based on professional inquiry and baseline examination. The participants and their parents or legal guardians were informed about the benefits and risks of this study before providing signed informed consent on behalf of their children.

### Interventions and visits

All spectacle lenses were made for single focus with full correction for each subject as the first intervention throughout the whole procedure for both groups. PBM therapy was performed with a low-intensity laser (LD-A, Jilin Londa Optoelectronics Technology, Jilin, China) with an irradiance of 0.35 ± 0.02 mW/cm^2^, a wavelength of 650 nm ± 10 nm, and illumination of approximately 400 lux on average.

During the baseline visit, eligibility was evaluated, and baseline measures were conducted. The dates of all subsequent visits were determined based on completion of the baseline examinations. Cycloplegic refraction using an automatic refractometer (Topcon KR-800, Topcon Corporation, Tokyo, Japan) and 100% subjective trial lenses were recorded at baseline and 12 months, respectively. Cycloplegia was achieved using 3 drops of 1% cyclopentolate hydrochloride eyedrop (CYCLOGYL, Alcon Laboratories, Inc., USA), and the spherical equivalent refraction (SER) was determined (obtained with the following formula: SER = spherical diopter + astigmatism/2). The follow-ups were conducted at 3 months, 6 months, 9 months and 12 months. The values of AL (IOLMaster 500, Carl Zeiss Meditec AG, Germany), CCP (Pentacam 70700, Oculus, Inc., Germany), SFChT (Spectralis OCT, Heidelberg Engineering GmbH, Germany) and ACD (Pentacam 70700, Oculus, Inc., Germany) were all recorded and evaluated at each visit in addition to the baseline visit.

Other ophthalmologic examinations included slit-lamp examination (HS-5000(HLG), Huvitz Co. Ltd, Korea), noncontact tonometry (Topcon CT-80, Topcon Inc., Japan), and fundus scan with optical coherent tomography (Spectralis OCT, Heidelberg Engineering GmbH, Germany).

### Evaluated parameters

The primary outcome variables were changes in AL and SER compared to baseline. SER (sphere plus half cylinder) from the pattern of five measurements was measured at least 30 min after instillation of 2 drops of 1% cyclopentolate administered every 5 min. AL was measured by calculating the average of five measurements obtained from the same IOLMaster. The secondary outcome variables included SFChT (Spectralis OCT, Herdingberg, Germany), ACD (from IOLmaster 500) and CCP (from Pentacam). PBM therapy was administered twice a day for 3 min per session with an interval of ≥ 4 h between sessions based on the self-report of participants or their supervision. We also evaluated the subjects’ social context, such as schools, and lifestyle (e.g., outdoor time) via questionnaires.

Due to the small sample size, we aimed to avoid the influence of age on different growth rates of AL. Therefore, the eligibility criteria for age were set from 8 to 12 years old. In addition, we also removed the limitation on baseline AL ≥ 24.40 mm because the myopia suppression effect is unknown for axial lengths greater than 24.40 mm.

### Sample size calculation

Power Analysis and Sample Size Software (PASS 2022) (NCSS, LLC. Kaysville, Utah, USA) was used to determine that the minimum sample size was 24. A previous study found that the rate of AL change was approximately 0.25 mm/year (0.13 mm/year vs. 0.38 mm/year) slower in participants treated with repeated low-level red light than in controls^5^. The mean AL progression was 0.13 mm and 0.38 mm with a standard deviation (SD) of 0.15 mm after 1 year in the control group based on previous findings^3,4^. This was based on a two-sided statistical test with a 1% type I error threshold, 90% power and a 20% drop-out rate.

### Randomization

Eligible participants enrolled in the study were assigned to receive single-vision spectacles (SVS) only or photobiomodulation (PBM) therapy with SVS at a 1:1 ratio using a randomization sequence produced by Excel. The mechanism used to implement the random allocation sequence was sequentially numbered containers by random envelopes. The enrolled subjects were randomly divided into two groups based on random numbers placed in envelopes. The random numbers were generated by Excel, and the aim was to achieve balanced treatment groups based on their baseline right-eye refractive error, mean age and gender. After all 50 participants were enrolled, the random envelopes were opened to indicate the group assignment. Researchers who were assessing outcomes and performers (for cycloplegic autorefraction and AL measurements) were still blinded to group allocation, but participants and care providers were not blinded after the random envelopes were opened.

### Adverse events

Those who received at least one session of therapy were analysed for safety. At each follow-up visit, the participants were asked about the symptoms and signs, including ocular symptoms (such as glare, dazzling, afterimages and flash blindness) and systemic adverse effects (such as headache or dizziness). Other information included the best corrected vision acuity (BCVA), anterior segment with slit-lamps and fundus with OCT. Additionally, each participant was also asked to report the reversible time of vision loss due to glare, dazzling, afterimages or flash blindness. Adverse events were reported based on interviews at the 12-month follow-up visit and were scored according to the common terminology standard for adverse events (CTCAE) version 5.0.

### Statistical analysis

The data were analysed by IBM SPSS statistical software 22.0 (IBM Co., Armonk, USA). Continuous variables were analysed with a two-sample independent t test after being evaluated for normality using the Kolmogorov‒Smirnov test. If the data conformed to a normal distribution, the mean ± standard deviation was reported. Fisher’s exact test or the χ^2^ test was conducted to compare the gender ratio between the two groups. ANOVA was used to compare the means between the baseline and follow-up visits. If the data were not normally distributed, the median (interquartile range) or median (95% confidence interval, 95% CI) was reported, and nonparametric tests were performed. A *P* value < 0.05 indicated significant differences. Only the data for the right eye were used.

The independent samples t test was used to analyse the changes in AL (as the primary endpoint), ACD, SFChT, and CCP at the 3-, 6-, 9-, and 12-month follow-ups as well as changes in cycloplegic refraction at the 12-month follow-up.

### Ethics approval and consent to participate

All procedures performed in studies involving human participants were in accordance with the ethical standards of the institutional and/or national research committee and with the 1964 Helsinki Declaration and its later amendments or comparable ethical standards. The study was approved by the Bioethics Committee of Ningbo Eye Hospital (No: 2020xjx-012).

### Conference presentation

This paper was presented at the conference of the 26th Congress of Chinese Ophthalmological Society (CCOS) 2022 and the World Ophthalmology Congress (WOC) 2022 online meeting.

## Results

**Figure 1:**
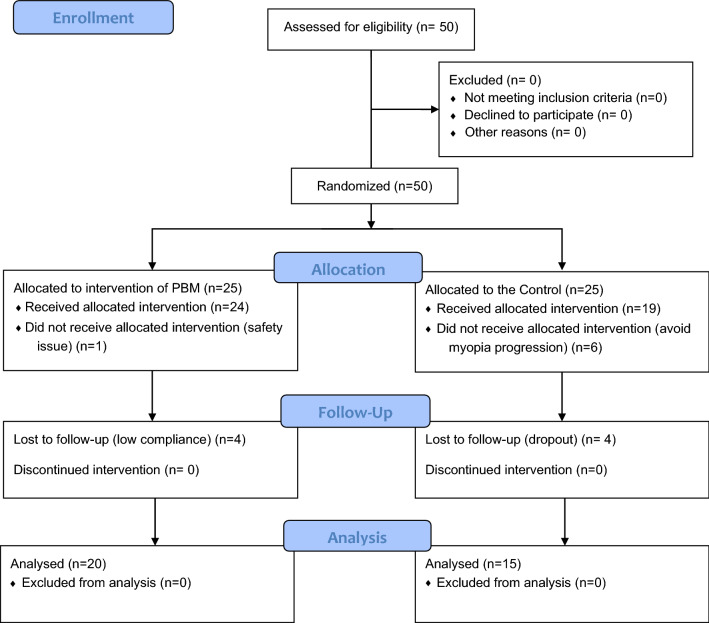
Flow diagram. *PBM* photobiomodulation therapy, *Control* single vision spectacles only.

From February to May 2021, 50 eligible subjects signed written informed consent forms before randomization, 43 subjects (24 in the PBM therapy group and 19 in the control group) completed the baseline visit, and 35 subjects completed the final 12-month follow-up visit (20 in the PBM group and 15 in the control group). The details of the participants are shown in Tables 1, 2 and 3. The flow diagram of the sample size and reasons for dropout are shown in Fig. 1.

**Table 1:** Baseline parameters of PBM and control groups.

	PBM group (n = 24)	Control group (n = 19)	*P*
AL (mm)	24.58 ± 1.16	24.38 ± 0.87	0.541^a^
SE (D)	-2.93 ± 1.87	-2.11 ± 1.21	0.105^a^
ACD (mm)	3.16 ± 0.26	3.30 ± 0.22	0.075^a^
SFChT (µm)	259.32 ± 54.68	239.77 ± 35.03	0.207^a^
CCP (D)	42.53 ± 1.30	42.56 ± 1.08	0.938^a^
Age (years)	10.17 ± 1.55	9.00 ± 1.20	0.008^a^
Gender (male:female)	15:9	10:7	0.818^b^

*AL* axial length, *SER* spherical equivalent of refractive error, *ACD* anterior chamber depth, *SFChT* subfoveal choroidal thickness, *CCP* central cornea power, *PBM group* photobiomodulation therapy group, *Control group* single-vision spectacles group. The *P* values were determined by the independent samples.

^a^t-test of the two groups, with the exception of gender, for which the ^b^Chi-square test was used.

**Table 2:** Changes from baseline measurements in ocular parameters for the two groups.

	3 Months (n = 43)			6 Months (n = 43)			9 Months (n = 35)			12 Months (n = 35)		
	PBM group	Control group	*P*	PBM group	Control group	*P*	PBM group	Control group	*P*	PBM group	Control group	*P*
Changes in AL (mm)	− 0.09 ± 0.07	0.14 ± 0.06	0.001	− 0.07 ± 0.09	0.23 ± 0.11	0.001	− 0.05 ± 0.06	0.36 ± 0.15	0.007	− 0.02 ± 0.11	0.48 ± 0.16	0.000
Changes in CCP (D)	− 0.06 ± 0.30	− 0.01 ± 0.27	0.608	− 0.01 ± 0.34	0.00 ± 0.13	0.147	− 0.02 ± 0.29	− 0.13 ± 0.28	0.457	0.16 ± 0.29	0.16 ± 0.36	0.980
Changes in ACD (mm)	0.24 ± 0.28	0.32 ± 0.30	0.396	0.02 ± 0.12	− 0.01 ± 0.07	0.498	0.05 ± 0.11	− 0.01 ± 0.24	0.071	0.04 ± 0.12	− 0.03 ± 0.16	0.170
Changes in SFChT (µm)	17.44 ± 42.37	11.09 ± 31.07	0.715	29.65 ± 50.34	20.60 ± 24.14	0.601	33.2 ± 54.59	− 5.5 ± 25.25	0.210	20.89 ± 39.48	11.14 ± 28.95	0.441
Changes in Cycloplegic SER (D)										+ 0.28 ± 0.26	− 0.97 ± 0.25	0.000

*AL* axial length, *CCP* central cornea power, *ACD* anterior chamber depth, *SFChT* subfoveal choroidal thickness, *PBM group* photomodulation group, *Control group* single-vision spectacles group, *SER cycloplegic* spherical equivalent refraction, *D* dioptre.

**Table 3:** Myopia control rates in absolute numbers (≤ baseline) and follow-up dropout rates.

	PBM therapy group	Control group	Effect size of the treatment for axial length (%)
Group baseline sample size	24	19	
Axial length control rate at 3 months	90.48%	0.00%	121.05
Axial length control rate at 6 months	77.27%	6.67%	130.43
Axial length control rate at 9 months	78.57%	16.67%	113.89
Axial length control rate at 12 months	60.00%	0.00%	103.33
Cycloplegic refraction control rate at 12 months	90.00%	0.00%	128.87
Dropout rate at 12 months	20.00%	40.00%	

PBM Therapy Group, Photobiomodulation Therapy Group; Control Group, single-vision spectacles group. The effect sizes of the treatment between the two groups were calculated with the following formula: $\frac{{\varvec{mean}}\; {\varvec{changes}}\; {\varvec{of}}\;{\varvec{PBM}}\;{\varvec{group}}-{\varvec{mean}}\; {\varvec{change}}\; {\varvec{of}}\;{\varvec{control}}\;{\varvec{group}}}{{\varvec{mean}}\; {\varvec{change}}\; {\varvec{of}}\;{\varvec{control}}\;{\varvec{group}}}$.

The characteristics of the baseline data are shown in Table 1. The total proportion of male participants was 61%, with a mean age of 9.7 ± 1.5 years. The cycloplegic SER of the right eyes ranged from − 0.75 to − 8.00 D with a mean value of − 2.56 ± 1.70 The mean AL, SER, ACD, CCP and SFChT did not differ based on any demographic variables except for age. The average age of the PBM therapy group was 1 year significantly older than that of the control group. The ocular characteristics (including AL, SER, ACD, CCP and SFChT) and sex were well balanced in both groups (Table 1). At baseline, no statistically significant difference was detected between the right and left eyes.

**Figure 2:**
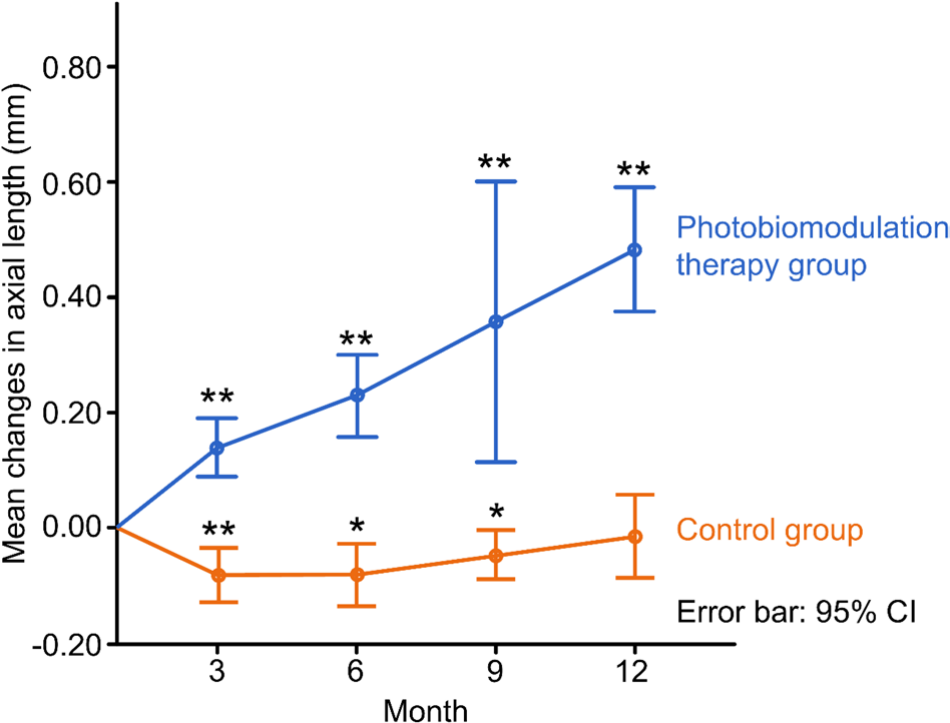
Changes of axial length of two groups at 3-, 6-, 9-, 12-month follow-up, respectively. ** and *represent *P* < 0.001 and *P* < 0.01 comparing the data of each follow-up with that of no change from baseline.

The mean change in AL decreased slightly in the PBM therapy group from baseline to the 12-month follow-up. In contrast, the mean change increased in the control group. When compared with the control group, the change in AL of the PBM group at each follow-up visit (3, 6, 9, and 12 months) was significantly better (− 0.09 ± 0.07 vs. 0.14 ± 0.06, *P* < 0.0001; − 0.07 ± 0.09 vs. 0.23 ± 0.11, *P* = 0.0001; − 0.05 ± 0.06 vs. 0.36 ± 0.15,* P* = 0.007; − 0.02 ± 0.11 vs. 0.48 ± 0.16, *P* < 0.0001, respectively), as shown in Table 2 and Fig. 2.

**Figure 3:**
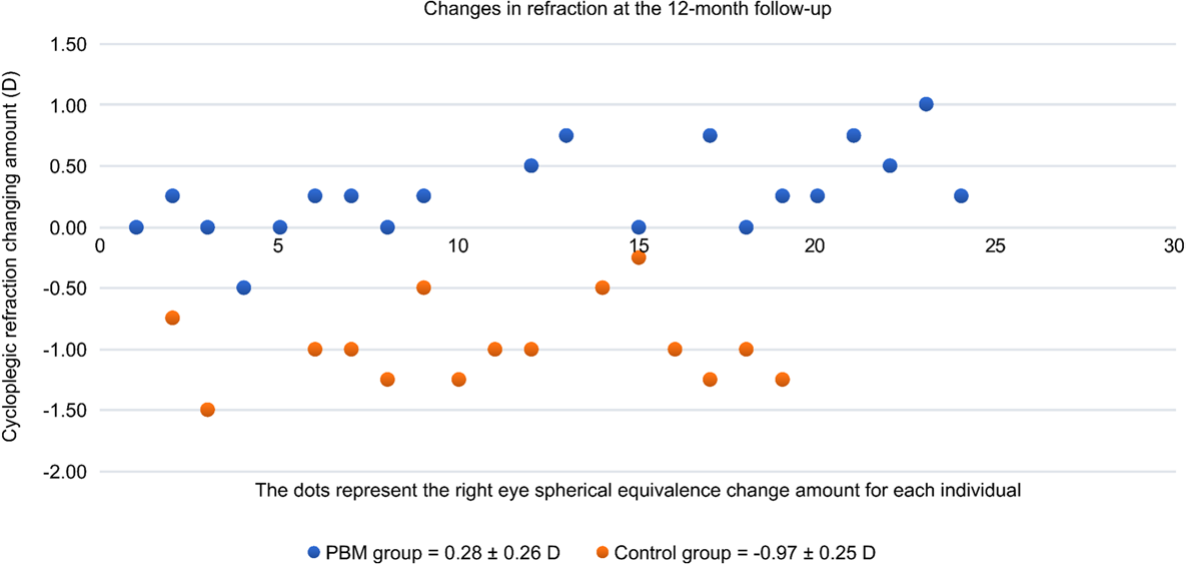
Changes in refraction individually at 12-month visit. *PBM* photobiomodulation therapy, *Control* single vision spectacles only.

Accordingly, the mean change in cycloplegic SER at 12 months from baseline was also significantly better in the PBM therapy group than in the control group (+ 0.26 ± 0.27 vs. − 0.97 ± 0.25, respectively, *P* < 0.0001). The mean SER value of the PBM therapy group decreased by approximately + 0.26 D, while the mean myopia of the control group progressed by − 0.97 D during the study (Fig. 3). The positive value (+ 0.26 D) of the mean SER change perfectly matched the negative change in the mean AL (− 0.02 mm), which explained the stabilization of myopia progression due to PBM therapy during the 12 months. The axial length of the control group increased, and the average calculated growth rate was − 2.02 D/mm (− 0.97 D/0.48 mm) during the 12 months.

There were no significant changes in any of the other variables, including CCP, ACD, and SFChT, between groups at any visit (Table 2). The myopia control rate and the dropout rate were calculated in terms of the AL and cycloplegic SER at the 12-month follow-up in both groups, and the results are shown in Table 3. The control rate was defined as an AL or SER at follow-up that was lower than the value at baseline. Similarly, the control rate of the PBM therapy group was also significantly higher than that of the control group. Specifically, the percentages of individuals who achieved AL and SER control were 60% and 90%, respectively, in the PBM group, while the percentages of individuals who attained AL and SE control were 0% and 0% in the control group. In addition, the withdrawal rate of the control group (40%) was twice as high as that of the PBM group (20%). Furthermore, neither social context, such as economic situations mainly by family income, nor lifestyle, such as near work and outdoor time, were significantly different between the two groups (Table 4).

**Table 4:** Results of questionnaires from the two groups.

Groups	Family income (RMB/month)	Nearwork time (minutes)/day	Outdoor time (hours)/day
PBM (n = 22)	24,077 ± 11,763 (N = 13)	62.95 ± 31.22	0.45 ± 1.86
Control (n = 22)	16,579 ± 9695 (N = 19)	69.63 ± 44.71	0.59 ± 1.57
*P* (t-test)	0.092	0.700	0.542

*PBM* photobiomodulation therapy, *Control* single vision spectacles group, *P* value was decided by the independent sample.

The most common adverse event was reversible vision loss, lasting approximately 2.1 ± 0.7 min (n = 23) after each PBM therapy due to flash blindness or glare with afterimage. In addition, afterimage, which lasted a little bit longer than the reversible vision loss—an average of 3.2 ± 1.2 min (calculated from the moment immediately lighting off of 650 nm PBM therapy)—was positive afterimage, i.e., central scotoma with different shapes in bright pink from the subjects after each PBM therapy (except the condition of closing the eyes). According to CTCAE (version 5.0), all cases were reversible and were all considered grade 1 eye disorders. All the above symptoms were subjective without objective abnormal findings from anterior or posterior ocular segments. There were no systemic adverse effects (such as headache or dizziness), severe adverse events, or other adverse events related to grades of ocular diseases: Grade 2–5 of CTCAE (version 5.0). Additionally, there was no dry eye, cataract, keratitis, night blindness, photophobia or any other permanent visual impairment.

There was one subject whose supervisor refused to join PBM therapy after randomization due to concern about safety issues, including the abovementioned temporary and reversible vision loss and afterimage phenomena. None of the reasons for dropout during follow-up in the PBM group were related to adverse events. An explanation was given for the different dropout rates between the control and PBM groups at baseline (6 vs. 3 dropouts, respectively) and at the 12-month follow-up (4 vs. 2 dropouts, respectively) (Fig. 1). After randomization, in the absence of additional intervention, the main reason for dropout in the control group was unacceptable (only SVS), whereas the main reason for dropout in the PBM group was understandable, as it was due to the uncertain harm of this new therapy. The COVID-19 pandemic was the main reason for the other missing data, especially at the 9-month visit, and both groups of patients were unable to visit regularly at that time.

## Discussion

As shown in Table 5, those results were consistent with our previous report and the studies of Xiong et al.^3,4,20^. It was shown that myopia among children could be controlled or reversed to some extent by PBM therapy, resulting in a mean decrease in the AL. Compared with baseline, this algorithm achieved 90% and 60% decreases in myopia based on SER and AL changes, respectively, instead of the values calculated based on the control group. However, because of the negative changes in AL and positive changes in SER, if the efficacy algorithm was based on the values from the control group with a formula of control rate = $\frac{{\varvec{Change}}\; {\varvec{of}}\;{\varvec{PBM}}\;{\varvec{group}}-{\varvec{Change}}\; {\varvec{of}}\;{\varvec{control}}\;{\varvec{group}}}{{\varvec{Change}}\; {\varvec{of}}\;{\varvec{control}}\;{\varvec{group}}}$*100%, both AL (103%) and SER (127%) control rates were > 100%. Jiang Y et al. used the same algorithm and observed yearly AL and SER control rates of 66% and 75%, respectively. The difference was probably due to their frequent administration of 5 times a day, including their reported low compliance.

**Table 5:** Changes in myopic axial length in studies of PBM therapy.

References	Study month (month)	Design	Number (n) (test/control)	Age (years)	Test group	Control group	AL changes (test/control, mm)	SER changes (test/control, D)
Xiong et al.^3^	6	RCT^a^	74/81/74	6–16	LLLT^b^	Ortho-k^c^/SVS^d^	− 0.06/0.06/0.23	+ 0.22/− 0.50
Jiang et al.^5^	12	RCT	117/129	8–13	RLRL^e^	SVS	0.13/0.38	− 0.20/− 0.79
Zhou et al.^4^	9	Retrospective cohort	105/NA^f^	4–14	RLRL	NA^f^	− 0.06/NA	+ 0.22/NA
Chen et al.^8^	12	RCT	51/51	6–13	LRL^g^	SFS^h^	0.01/0.39	+ 0.05/− 0.64
Dong et al.^6^	6	RCT	56/55	7–12	RLRL	Sham device	0.02/0.13	+ 0.02/− 0.11
Xiong et al.^7^	24	RCT	41/10/52/11	8–13	RLRL-RLRL	SVS-RLRL/RLRL-SVS/SVS-SVS	0.12/0.05/0.42/0.28	− 0.20/− 0.09/− 0.91/− 0.54
Yi et al.^11^	12	RCT	66/66	7–12	1%Atropine	Placebo	− 0.03/0.32	0.32/− 0.85
Ruiz-Pomeda, et al.^13^	24	RCT	46/33	8–12	Misight	Disposal single vision contact lenses	0.28/0.44	− 0.45/− 0.74
Yam et al.^15^	12	RCT	110/110/110	4–12	0.05%atropine/0.025%atropine/0.01%atropine	Placebo	0.20/0.29/0.36/0.41	− 0.27/− 0.46/− 0.59/− 0.81
Yam et al.^16^	24	RCT	383	4–12	0.05%atropine/0.025%atropine/0.01%atropine	Placebo	0.39/0.50/0.59	− 0.55/− 0.85/− 1.12
Cho et al.^19^	24	RCT	37/41	6–10	Ortho-K lenses	Single-vision glasses	0.36/0.63	NA
Wang et al.^20^	12	Retrospective cohort	434/NA	3–17	RLRL	NA	− 0.142/NA	NA
Chen et al.^21^	12	RCT	31/31	7–15	RLRL	0.01% atropine	0.08/0.33	− 0.03/− 0.60

^a^*RCT* randomized control trial, ^b^*LLLT* low level light therapy, ^c^*Ortho-K* orthokeratology, ^d^*SVS* single vision spectacles, ^e^*RLRL* repeated low-level red light, ^f^*NA* not applicable, ^g^*LRL* low-intensity red-light, ^h^*SFS* single focus spectacles.

As one of the most effective interventions compared with other interventions^12–18,20,21^, we found evidence supporting our hypothesis that the progression of myopia as measured by the AL is reversible using PBM therapy twice a day.

All the negative mean changes in AL from the study group at 3, 6, 9, and 12 months (− 0.09 ± 0.07 mm, − 0.07 ± 0.09 mm, − 0.05 ± 0.06 mm and − 0.02 ± 0.11 mm) demonstrated that PBM could retard or control increases in AL. In contrast, all the positive mean changes in AL from the control group at each visit (0.14 ± 0.06 mm, 0.23 ± 0.11 mm, 0.36 ± 0.15 mm, 0.48 ± 0.16 mm at 3, 6, 9, and 12 months, respectively) revealed a strong natural tendency towards increased AL among primary school-age children. Although the age of the control group was significantly younger by 1 year, the decrease in the mean values of AL in the PBM therapy group also indicated that the administration with a frequency of twice daily in our trial was better than the frequency of five times a week in Jiang Y et al.’s study with an mean AL increase of 0.13 mm per year^5^. In addition, in past prospective myopic interventions, almost no negative change in mean AL per year was reported, and the only comparable effect was 1% atropine treatment. As reported by Yi S et al. the negative average change in the mean AL was − 0.03 ± 0.30 mm^11^. Balancing the pros and cons, PBM therapy had much less photophobia, much shorter blurred vision time and much fewer other AEs than 1% atropine therapy^11^ in our study or repeated low-level red light (RLRL) therapy^5,7^. The other optical interventions are partly shown in Table 5, such as Misight myopia control disposal soft contact lenses^13,14^, different low concentrations of atropine eyedrops^12,15,16^, innovative spectacle lenses with D.I.M.S. technology^17,18^, or orthokeratology, as reported by Dr. Pauline Cho, with a 43% myopia control rate compared to single-vision glasses^19^. However, participants in that study still had positive annual AL value changes.

The mechanism underlying the negative values of changes in AL is unclear since there were no other parameters studied herein, such as SFChT, ACD, or CCP, that could reasonably explain this phenomenon.

The first RCT with a 6-month follow-up on PBM intervention in children with myopia reported a statistically significant thicker SFChT^3^, while our results revealed no statistically significant difference between the two groups due to the accuracy of OCT choroidal thickness and the limited sample size. According to our experimental data, 65% of the individuals with increased SFChT in the PBM group reported a decreased AL at the last visit, while only 36% of the individuals with thinner SFChT in the control group reported an increase in AL during the same period. It remains unclear whether the changes in SFChT can explain the changes in AL because PBM therapy was defined as the utilization of nonionizing electromagnetic energy to trigger photochemical changes within cellular structures that absorbed photos. Mitochondria are particularly sensitive to this process, with more ATP for cellular use with a key enzyme called cytochrome oxidase C. In addition, that process produces mild oxidants (ROS), leading to gene transcription, which then leads to cell repair and healing. This process can also remove the chains clogged by nitric oxide (NO).

NO is a molecule produced by our body that helps the 50 trillion cells in our body communicate with each other through signalling, helping to dilate blood vessels, and improving blood circulation. Our vision is based on light hitting our retina and generating a chemical reaction that enables us to see.

Regarding vision, none of the participants had reduced BCVA. BCVA was the inclusive criterion for enrolment in the current study, and there was no special record to compare the differences between the two groups. However, some studies have reported significantly better vision after PBM therapy^5^.

In view of the limitations of this study, a non-double-blind study was designed. We did not provide sham devices for the control group, thus avoiding the subjective tendency of the treatment or placebo effect. Additionally, there was a high withdrawal rate at baseline in the control group after randomization, which disrupted the balance in the ages of the two groups. The significant bias created by having a significantly younger control group at baseline. The mean age of the PBM group (mean age: 10.2 ± 1.6 years) was older than that of the control group (mean age: 9.0 ± 1.2 years), and it was known that axial length increases faster in younger people. Although at 9-month visit, the mean ages from the two groups (9.9 ± 1.4 years, vs. 8.4 ± 0.5 years) were not statistically significant (*P* = 0.07).

Another limitation was the limited sample size and the slightly high dropout rate, especially in the control group. The limited number of samples might have explained the lack of a significant difference in SFChT or ACD, since the calculation of sample number was based on the AL only.

Future research should explore the different sources of indoor light, such as light emitting diode (LED), or related parameters, such as laser output energy, radiation wavelength, exposure duration and laser beam cross-sectional area of the point of interest. Additionally, different forms of administration in the clinic should be examined.

There is still a long way to go to determine the optimal phototherapy parameters for myopia or other related diseases. Long-term prospective programs are needed to identify the optimal conditions. In addition, the long-term effect of this technology remains unclear.

Briefly, the current 12-month study showed that PBM is a new, noninvasive and safe intervention with higher efficacy and safety for controlling, delaying and reversing the progression of myopia in children.

## Conclusions

PBM therapy is an effective treatment to delay and retard increases in AL among children with myopia; this therapy is accompanied by approximately 2.1 min of temporary vision loss.

## Data Availability

Datasets of this study are available from the corresponding authors upon reasonable request.
